# Peroxynitrous Acid Generated In Situ from Acidified H_2_O_2_ and NaNO_2_. A Suitable Novel Antimicrobial Agent?

**DOI:** 10.3390/antibiotics10081003

**Published:** 2021-08-19

**Authors:** Martina Balazinski, Ansgar Schmidt-Bleker, Jörn Winter, Thomas von Woedtke

**Affiliations:** Leibniz Institute for Plasma Science and Technology, Felix-Hausdorff-Straße 2, 17489 Greifswald, Germany; ansgar.schmidt-bleker@inp-greifswald.de (A.S.-B.); winter@inp-greifswald.de (J.W.); woedtke@inp-greifswald.de (T.v.W.)

**Keywords:** peroxynitrite, peroxynitrous acid, *E. coli*, Ames, dose-response relationship

## Abstract

Peroxynitrite (ONOO^−^) and peroxynitrous acid (ONOOH) are known as short acting reactive species with nitrating and oxidative properties, which are associated with their antimicrobial effect. However, to the best of our knowledge, ONOOH/ONOO^-^ are not yet used as antimicrobial actives in practical applications. The aim is to elucidate if ONOOH generated in situ from acidified hydrogen peroxide (H_2_O_2_) and sodium nitrite (NaNO_2_) may serve as an antimicrobial active in disinfectants. Therefore, the dose-response relationship and mutagenicity are investigated. Antimicrobial efficacy was investigated by suspension tests and mutagenicity by the Ames test. Tests were conducted with *E. coli*. For investigating the dose-response relationship, pH values and concentrations of H_2_O_2_ and NaNO_2_ were varied. The antimicrobial efficacy is correlated to the dose of ONOOH, which is determined by numerical computations. The relationship can be described by the efficacy parameter *W*, corresponding to the amount of educts consumed during exposure time. Sufficient inactivation was observed whenever *W* ≥ 1 mM, yielding a criterion for inactivation of *E. coli* by acidified H_2_O_2_ and NaNO_2_. No mutagenicity of ONOOH was noticed. While further investigations are necessary, results indicate that safe and effective usage of ONOOH generated from acidified H_2_O_2_ and NaNO_2_ as a novel active in disinfectants is conceivable.

## 1. Introduction

Disinfectants play an important role in our society, especially with the accelerated growth of the economy and increasing globalization. Most disinfectants for skin use are based on alcohol and these are recommended by the WHO Guideline [1]. They are effective against various bacteria, fungi, and viruses, but they show a lack in sporicidal efficacy [2,3].

A possible candidate for a surface and skin disinfectant is peroxynitrous acid (ONOOH) synthesized in situ from acidified hydrogen peroxide (H_2_O_2_) and nitrite (NO_2_^−^). ONOOH is known for its strong antimicrobial properties, which appear to be associated with membrane damage [4,5]. Cell biological analysis shows interactions of ONOO^−^ with DNA, lipids, and proteins. A relation to direct oxidative reactions or indirect, radical-mediated mechanisms can be found. These various reactions and mechanisms can further be attributed to various pathological incidents in humans [6,7].

Under physiological conditions at pH 7.4, its conjugated base peroxynitrite (ONOO^−^, pK_s_ 6.8) is produced from NO and O_2_^−^ as part of the innate immune response [8]. In acidic conditions, ONOOH can be synthesized via the net reaction (the reaction is likely to proceed via a two-step process involving the formation of H_3_O_2_^+^ [9]).

H_2_O_2_ + HNO_2_ → ONOOH + H_2_O. (R1)

with pH-dependent reaction coefficient according to Vione [9],

$$k_{R1}=\frac{179.6\times {\left(10^{-\mathrm{pH}}\;\right)}^{2}}{\left(3.4\times 10^{-2}+10^{-\mathrm{pH}}\right)\;\left(6\times 10^{-4}+10^{-\mathrm{pH}}\right)\;}.$$

Its oxidating and nitrative properties arise from ONOOH itself or from reactions of its decay products, the hydroxyl radical (OH^•^) and nitrogen dioxide (NO_2_^•^) arise according to the reactions

ONOOH → NO_2_^•^ + OH^•^ (30%), (R2)

ONOOH → H^+^ + NO_3_^−^ (70%). (R3)

with the indicated branching ratios [10].

While ONOO^−^ is stable under clean conditions in alkaline solution (in practice, ONOO^−^- solutions are stored at −80 °C) [11], the lifetime of ONOOH ($\tau_{\mathrm{ONOOH}}$) due to reactions R2 and R3 is 0.90 s [12]. In physiological solution, e.g., at pH 7.4 [13], ONOOH is mainly present as the conjugated base ONOO^−^ due to its pK_s_ value of 6.8 [14]. However, in physiological media, its lifetime is in the order of ms due to the presence of CO_2_ [15].

ONOO^−^ and ONOOH have previously been investigated for disinfecting bulk liquids and biofilms, e.g., in wastewater treatment [16], disinfecting contact lenses, and in case of bioterrorism contamination events [17,18]. Furthermore, it is discussed to be a major antimicrobial component of aqueous liquids treated with physical plasma, in which H_2_O_2_ and HNO_2_ (among other species) are continuously generated [19,20].

However, ONOOH-based disinfection has not become established by now. It is likely that the short-lived nature and reactivity of ONOOH makes its use difficult.

A direct measurement of ONOOH is difficult due to its short lifetime, spontaneous decay, and high reactivity. Measurements of ONOOH are obtainable by performing fluorescent measurements with 2,7-dichlorodihydrofluorescein diacetate as fluorescence dye [21]. Due to the experimental setup, the mentioned method is not suitable because in acidified conditions only a small fluorescence signal is detectable [22].

**Figure 1:**
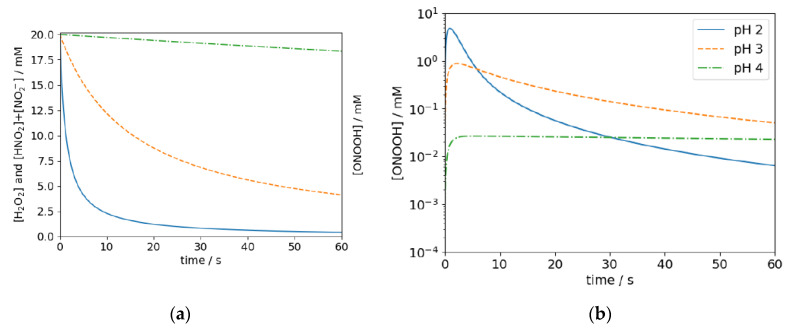
Time dependence of the concentration of the educts H_2_O_2_ and HNO_2_ (**a**) and resulting concentration of ONOOH at pH 2, 3, and 4 (**b**). The data is obtained by numerically solving the rate equations for reactions R1, R2, and R3.

Furthermore, generated in situ by mixing acidified H_2_O_2_ and NaNO_2_, the concentration of both the educts and ONOOH itself are strongly time-dependent as illustrated in Figure 1. The exemplary data was obtained by solving the rate equations for reactions R1, R2, and R3 numerically.

The concentration of ONOOH at pH 2 rapidly increases when the reactants are mixed but drops by three orders within a minute. In effect, most of the educts may already be consumed and ONOOH may have decayed when the mixture reaches its target in a practical disinfection process. Hence, it is yet unclear if ONOOH-based disinfectants can be applied for practical disinfection procedures in which processing times in the range of tens of seconds to several minutes are required, e.g., the distribution and the diffusion on or into potentially uneven or porous surfaces.

Furthermore, while several in vitro investigations on the mutagenicity of ONOO^−^ have been conducted, to this day, little is known about the mutagenic potential of ONOOH [23,24,25,26,27].

In this work, the efficacy against bacteria and the mutagenic potential of ONOOH generated in situ from H_2_O_2_ and NO_2_^−^ are studied using *E. coli* as a model microorganism. The data have partially been published previously as part of a patent document and shall be presented here to the scientific community in the required level of detail [28].

The antibacterial efficacy is investigated using the quantitative suspension test considering a waiting time of 30 s after mixing the educts H_2_O_2_ and NaNO_2_ (mimicking real-world processing and diffusion times) at various pH levels ranging from 2.4 to 5.9. Because practical applications such as surface and hand disinfection require disinfectants to be effective under high organic load conditions, CASO broth is added during the suspension tests. The hygienic hand rub tests also employ proteins as protein load [29]. The dose-response relationship is studied in detail by comparing various pH and H_2_O_2_/NO_2_^−^ concentrations to the achieved antimicrobial efficacy.

It is shown that the antimicrobial efficacy of acidified H_2_O_2_ and NO_2_^−^ against *E. coli* correlates to the proposed efficacy parameter W, which corresponds to the amount of educts ([H_2_O_2_] ≙ [HNO_2_/NO_2_^−^]) consumed during the exposure time: If *W* > 1 mM, more than 1 mM H_2_O_2_ or HNO_2_/NO_2_^−^ are consumed during exposure time, a sufficient inactivation (>99.99%) is achieved. This, for the first time, allows a layout of an ONOOH-based disinfectant for a target application by employing a priori numeric calculations.

Relating to its possible use as a disinfectant, the mutagenicity of ONOOH is of high interest. The Ames test is a method that gives a hint toward a mutagenic potential. Studies were previously published on ONOO^−^ [30]. In our studies, the Ames test was performed at acidic pH, preventing the formation ONOO^−^.

## 2. Materials and Methods

### 2.1. Microorganisms and Culture Conditions

For the antimicrobial testing the microorganism *E. coli* DSM 11250 (DSM-German Collection of Microorganisms and Cell Cultures, Braunschweig, Germany) was used for suspension tests. The microorganism was cultured on CASO-agar plates (Carl Roth GmbH & Co. KG, Karlsruhe, Germany). After incubation for 24 h at 37 °C, plates were stored at 8 °C. For the experiments, one colony was transferred to 25 mL CASO broth (Carl Roth GmbH & Co. KG, Karlsruhe, Germany) and cultured as an overnight culture (20 h, 37 °C). After the incubation time, 10 mL of the culture was centrifuged for 5 min (4500 rpm, Heraeus Multifuge 1S, Thermo Fisher Scientific, Dreieich, Germany). The supernatant was discarded, and the cells were suspended in 10mL of saline solution (0.85% NaCl (Carl Roth GmbH & Co. KG, Karlsruhe, Germany)). The bacteria suspension was adjusted to a total viable count of approximately 8 log_10_ cfu/mL (cfu: colony forming unit; 10^8^ cfu/mL, stock suspension).

For the implementation of the Ames test, *E. coli WPA2* with a mutation at the trpE gene (DSM 9495) was chosen. This microorganism was cultured on minimal glucose agar plates as described [31].

### 2.2. Suspension Tests with Various pH and H_2_O_2_/NO_2_^−^ Concentrations

ONOOH was formed by mixing solutions of H_2_O_2_ and NaNO_2_ at pH values ranging from 2.4 to 5.9 and equimolar initial concentrations of H_2_O_2_ and NO_2_^−^ of 0.05, 0.25, 0.5, 2.5, 5, 10, 25, 20, and 30 mM, respectively.

The suspension test consisted of five steps:

1. 220 µL of CASO broth and 100 µL of *E. coli* stock suspension (1:100 dilution) were transferred to 1.5 mL Eppendorf cups.
2. In a further Eppendorf cup, 390 µL of a NaNO_2_ solution was prepared and 390 µL of phosphate-citrate buffer solution (see section below) containing H_2_O_2_ was added.
3. After 30 s of waiting, the mixture was added to the cup containing *E. coli* as prepared in step 1.
4. The bacteria solution was exposed to ONOOH generated from NaNO_2_ and H_2_O_2_ for an exposure time of approximately 15 to 20 s.
5. 50 µL of the solution were plated in logarithmic order on agar plates (Eddyjet 2, I&L Biosystems GmbH, Königswinter, Germany).

The subsequent counting of the colonies was conducted with a colony counter (Flash&Go, I&L Biosystems, Königswinter, Germany). The negative control experiments were performed by employing distilled water instead of buffered H_2_O_2_ and NaNO_2_-solution in the procedure described above. The evaluation of the reduction of the microorganism *E. coli* was depicted by the calculation of the reduction factor according to

(1) $$\mathrm{reduction}\;\mathrm{factor}=\log_{10}(N_{\mathrm{control}})-\log_{10}(N_{\mathrm{treatment}}),$$

where *N*_control_ and *N*_treatment_ are the cfu of the control and of the treated microorganisms, respectively.

The buffer solutions employed in the suspension tests were designed based on the McIlvaine buffer [32]. Buffer solutions in the range from pH 2.4 to 5.9 were prepared from a 450 mM sodium hydrogen phosphate (Na_2_HPO_4_) solution (Na_2_HPO_4_ ∙ 2 H_2_O, Carl Roth GmbH & Co. KG, Karlsruhe, Germany) and a 1000 mM solution of citric acid (citric acid, anhydrous, Carl Roth GmbH & Co. KG, Karlsruhe, Germany). The measured pH values along with the corresponding pH values used in the numerical calculations are shown in Table 1. During the suspension tests, the buffer was mixed with H_2_O_2_ solution (H_2_O_2_ *w* = 30%, Merck KGaA, Darmstadt, Germany), NaNO_2_, and CASO- broth (both Carl Roth GmbH & Co. KG, Karlsruhe, Germany) in the mixing ratios indicated above. In order to ensure that the buffer is strong enough to maintain the desired pH within ±0.1 pH units during the experiments, the pH was measured after step 2 and after step 3 of the procedure described above. The measurements after each step were mainly established to estimate the impact of the addition of CASO broth on the pH value. The indicated concentrations of H_2_O_2_ and NaNO_2_ referred to the respective initial concentrations in the mixture obtained in step 2. The pH values per row shown in Table 1 increase by shifting the proportion of Na_2_HPO_4_ to citric acid for the benefit of Na_2_HPO_4_ (sample 1 = lowest proportion of Na_2_HPO_4_, sample 8 = highest proportion of Na_2_HPO_4_). The adjustment process was created manually by the usage of the pH electrode (Mettler Toledo Seven Excellence Multiparameter, Gießen, Germany) to ensure the correctness of the pH employed in numerical calculations.

### 2.3. Evaluation of Efficacy Parameter

It is assumed that the dose-response relationship can be described using the Haber’s efficacy parameter *H* corresponding to the temporal integral over the concentration of ONOOH during the exposure time ranging from *t*_0_ to *t*_1_, where *t*_0_ is the time point when ONOOH is first in contact with the bacteria and *t*_1_ resembles the endpoint of the disinfection process:

(2) $$H=\int_{t_{0}}^{t_{1}}\left[\mathrm{ONOOH}\right]\;dt.$$

However, in most practical applications, the time-dependent concentration of ONOOH is not accessible. As recommended by Schmidt-Bleker et al. [28], it is favorable to define the efficacy parameter W as the total concentration of ONOOH generated during the exposure time:

(3) $$W=\int_{t_{0}}^{t_{1}}R\left(t\right)\;dt,$$

where R(*t*) is the production rate of ONOOH

(4) $$R\left(t\right)=k\left(pH\right)\left[HNO_{2}\right]\left[H_{2}O_{2}\right],$$

according to reaction R1 with the pH-dependent reaction coefficient *k* [9]. When the lifetime of ONOOH is much shorter than the exposure time *t*_1_ − *t*_0_,

(5) $$W\approx \frac{H}{\tau_{\mathrm{ONOOH}}},$$

**Figure 2:**
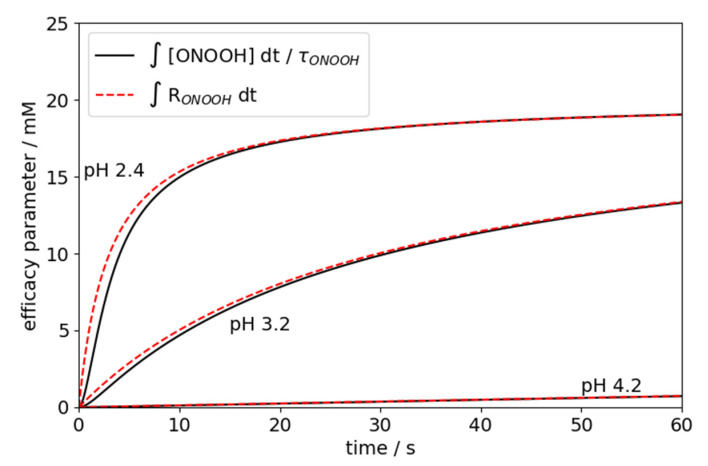
Exemplary comparison of the indefinite integrals according to Haber’s efficacy parameter (see Equation (1)) and the efficacy parameter W (Equation (2)).

This is demonstrated in Figure 2 in which the computed indefinite integral over R(*t*) in Equation (3) is compared to the computed efficacy parameter according to Haber (Equation (2)). It can be observed that slight deviations between *H*/$\tau_{\mathrm{ONOOH}}$ and W can only be expected for extremely fast processes. The advantage of employing the parameter W is that it equals the total amount of total nitrite (HNO_2_ + NO_2_^−^) and H_2_O_2_ that have reacted during the exposure time (as H_2_O_2_ and HNO_2_ are consumed at equal amounts according to reaction R1, *W* can be determined from the consumption of either species), e.g.,

(6) $$W=\left[H_{2}O_{2}\right]\left(t_{1}\right)-\left[H_{2}O_{2}\right]\left(t_{0}\right)=\left[HNO_{2}\right]\left(t_{1}\right)+\left[NO_{2}^{-}\right]\left(t_{1}\right)-\left[HNO_{2}\right]\left(t_{0}\right)-\left[NO_{2}^{-}\right]\left(t_{0}\right),$$

which, in contrast to ONOOH, may often be measurable using test strips or employing UV spectroscopy.

In the frame of this work, W was computed numerically using SciPy [33]. In order to account for the dilution in step 3 of the suspension test described in Section 2.2. the concentrations were computed in two steps: In the first step, the concentrations were computed in the interval (0, *t*_0_). Then, all concentrations were multiplied with the factor 0.71 (780 µL reaction mass of H_2_O_2_ and NO_2_^−^ divided by 1100 µL total volume), using the computed concentrations as starting values for the computation of the concentrations during the exposure time (*t*_0_, *t*_1_).

### 2.4. Ames Test

For the testing of a mutagenic effect of the mixture of H_2_O_2_ and NO_2_^−^ solutions, an Ames test was conducted. The method was adopted and performed as a plate incorporation assay [31]. As a negative control, ultrapure water (Thermo Fisher Scientific GenPure Pro Barnstead, Dreieich, Germany) was used. The positive control was achieved by applying the chemical 4-Nitroquinoline-*N*-oxide (VWR International GmbH, Hannover, Germany), which is a known mutagenic agent. The test sample was obtained by mixing 50 mM NaNO_2_ solution with a solution containing 50 mM H_2_O_2_ containing 2% citric acid. In order to obtain diluted samples of the test sample, both solutions were diluted separately prior to mixing. Amounts of 100 µg, 10 µg, 0.1 µg 4-Nitroquinoline-*N*-oxide, and 0 µg as control, respectively, were added to the molten top agar and poured on the agar plates. In total, the molten top agar was composed of 100 µL of a 9 log_10_ cfu/mL overnight culture of *E. coli* WP2, 10–200 µL of 4-Nitroquinoline-*N*-oxide (accordingly to concentration added with distilled water up to 200 µL), or 200 µL of the test sample (100 µL of each solution). For pH stabilization, 500 µL sodium phosphate buffer solution was included.

## 3. Results and Discussion

### 3.1. Suspension Tests with Various pH and H_2_O_2_/NO_2_^−^ Concentrations

**Figure 3:**
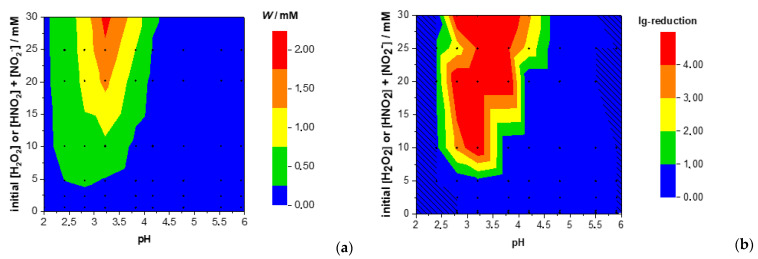
Measured log_10_ reduction factors of *E. coli* of individual experiments (**a**) at indicated pH and initial [H_2_O_2_] ≙ [HNO_2_ + NO_2_^−^] and (**b**) computed efficacy parameter W at indicated pH and initial [H_2_O_2_] ≙ [HNO_2_ + NO_2_^−^].

The inactivation rate of microorganisms was investigated in dependence of varying pH values and concentrations of H_2_O_2_ and NO_2_^−^ solutions. The experiments were performed in three sets with separate negative control experiments. The untreated control experiments yielded 8.06 ± 0.04, 7.36 ± 0.04, and 8.32 ± 0.05 log_10_ cfu/mL. The results of the means of the reduction factors, and accordingly, the computed efficacy parameter *W*, both at indicated pH and initial [H_2_O_2_] ≙ [HNO_2_+ NO_2_^−^], are illustrated in Figure 3.

**Figure 4:**
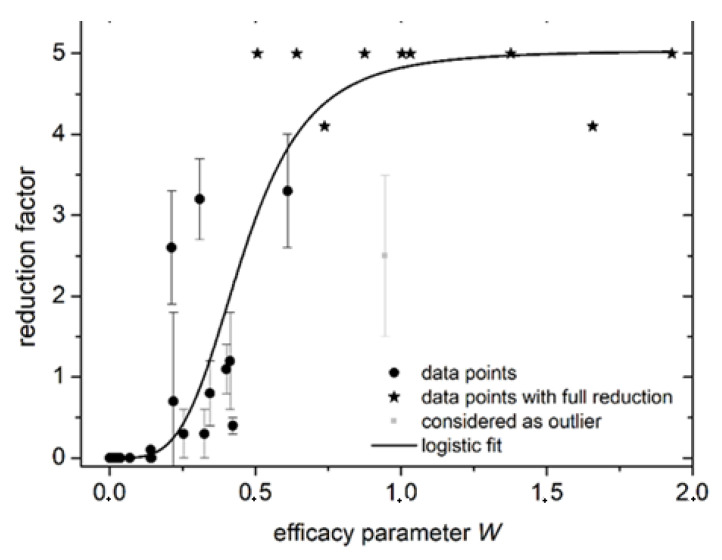
Scatter plot of reduction factor and efficacy parameter W.

Figure 3a shows that the log_10_-reduction is maximal at concentrations of ~10 mM–30 mM within a pH of 3.25. At higher or lower pH values the log_10_-reduction decreases. For concentrations less than 5 mM, little to no reduction can be achieved at any pH. The same is true for pH values greater than 4.75. In Figure 3b, the computed efficacy parameter W, calculated according to equation 3 for all pH and concentration combinations of Table 2 is presented. The shape of Figure 3a is well comparable to Figure 3b. To clarify this point, the relation between reduction factor and efficacy parameter is displayed in Figure 4.

**Figure 5:**
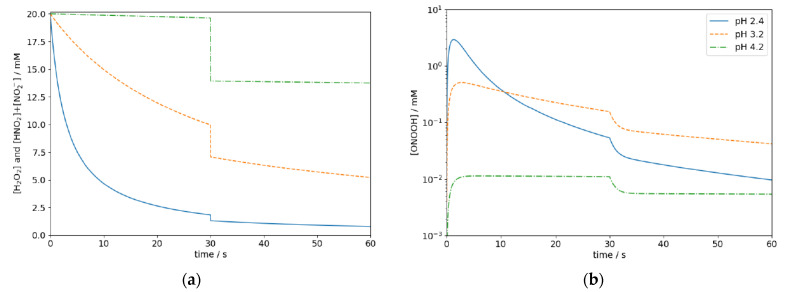
Computed concentrations of H_2_O_2_ ≙ HNO_2_ + NO_2_^−^ and (**a**) ONOOH (**b**) at pH 2.4, 3.2, and 4.2 at initial concentration of H_2_O_2_ ≙ HNO_2_ + NO_2_^−^ of 20 mM.

The shape of the efficacy map in Figure 3b can easily be understood by examining Figure 5, which shows the computed time-dependent concentrations of H_2_O_2_ and HNO_2_ + NO_2_^−^ and ONOOH at three exemplary parameters.

At low pH of 2.4, the reaction R1 proceeds too fast, and hence too little educts are left after the 30 s waiting period to sufficiently inactivate the bacteria during the exposure time (*t*_1_ − *t*_0_). At pH 4.2, the reaction proceeds too slow, while at pH 3.2, the highest ONOOH concentration can be expected during the exposure time.

The antimicrobial efficacy of ONOOH generated from acidified H_2_O_2_ and NaNO_2_ toward *E. coli* correlates to the dose (concentration × exposure time) of ONOOH. The efficacy can be described using a simplified efficacy parameter W, which corresponds to the amount of H_2_O_2_ or HNO_2_ + NO_2_^−^ consumed during the exposure time. A log_10_ reduction of ≥4 is obtained whenever W ≥ 1 mM. The inactivation was achieved in a solution containing CASO broth, which shows that a ONOOH-based decontamination may work under conditions of high organic load. The optimum pH values of 3–4 and initial concentrations of NaNO_2_ and H_2_O_2_ greater than 10 mM will depend on the employed waiting time *t*_0_, indicating the time between the mixing of educts and the time of application. Furthermore, an efficacy parameter of 1 mM may depend on the given organic load, as this may affect the lifetime of ONOOH in the solution. It is observed that neither a low pH of 2.4 alone nor high concentrations of H_2_O_2_ ≙ HNO_2_ + NO_2_^−^ of 30 mM are sufficient to inactivate *E. coli* during an exposure time of 30s. Previous publications that investigated the antimicrobial efficacy of plasma-activated water also indicate peroxynitrous acid to be the most important reactive oxygen and nitrogen species (RONS) for elimination of bacteria [34]. Furthermore, acidified nitrates and H_2_O_2_ did not show any lethal effect when applied separately, whereas a synergistic lethal effect was predicted when chemical compounds were mixed [35].

### 3.2. AMES Test

The mutagenetic tests were conducted with a fixed concentration of 25 mM H_2_O_2_ and 25 mM NO_2_^−^, in mixture. The concentrations used were set to a lower level than presented in the experimental part, as the test solution shows bactericidal effects and higher concentrations will probably lead to no growth because of complete elimination. Furthermore, the test requires to be conducted at the highest non-toxic concentration of the test product [36]. Since elimination can hide possible mutagenic properties, different tests are necessary to determine the mutagenicity at higher concentrations of the test product. The used mutagenic agent as positive control was 4-Nitroquinoline-*N*-oxide in various concentrations (0.1, 10, and 100 µg per plate). Ultrapure water was used as negative control. In Table 2 the resulting cfu per plate are listed when either treating the *E. coli* WP2 strain with the test product for 30 s before plating or incubating the bacteria on plates equipped with 4-Nitroquinoline-*N*-oxide as positive control. The incubation time for positive control and test product was 17 h.

For the test product, 100% corresponds to an initial concentration of 25 mM NaNO_2_, 25 mM H_2_O_2_, and 1% citric acid in the mixed solution. For the positive control, 100% corresponds to 100 µg 4-Nitroquinoline-*N*-oxide per plate. In this test, high numbers of cfu per plate show a high mutagenic activity. The number describes the amount of occurring revertants. These are mutants that have reverted to its origin by a further occurring mutation. In this case, the revertants regain the ability to metabolize the existing tryptophan in the agar. This process is triggered naturally or by mutagenic agents. The negative control with ultrapure water results in 7.0 ± 1.2 cfu/plate, whether or not the positive control shows a significant increase on cfu/plate up to 170 by a concentration of less than 1% per plate of 4-Nitroquinoline-*N*-oxide. For the test product, no significant increase is observed at concentrations ranging from 0.4% to 10%. The number of colonies observed for the test products at concentrations ≤ 10% suggest naturally occurring revertants due to the same level of revertants in comparison to the negative control. At higher concentrations, the test product led to a complete inactivation of the bacteria. As the complete inactivation can be a result of either antibacterial efficacy of the test product or a mutagenic effect, no clear result can be concluded on the mutagenic potential of the test product in this case [37].

## 4. Conclusions

The efficacy parameter W corresponds to the amount of educts (H_2_O_2_ or HNO_2_) consumed at a given pH value during the exposure time and produces a clear criterion for the inactivation of *E. coli* by ONOOH. If W > 1 mM, a sufficient inactivation can be expected. This leads to a better understanding of the reaction kinetics and allows a precise design for further experiments. In future work, the efficacy parameter should be determined for other microorganisms.

Regarding the AMES test, the findings do not hint toward a possible mutagenic potential of ONOOH for concentrations of ≤25 mM H_2_O_2_ and NaNO_2_. Further testing is necessary to assess the mutagenic potential of ONOOH for higher concentrations. These can be conducted by using mammalian cells [38].

In conclusion, in situ generated ONOOH from acidified H_2_O_2_ and NO_2_^−^ may serve as a disinfectant for practical purposes. The investigations on the antimicrobial efficacy incorporate well toward the findings of several studies, and peroxynitrous acid is to play an important role in the elimination of bacteria [34]. However, particular attention must be paid to the required processing and exposure times, as the rapid degradation of H_2_O_2_ and HNO_2_ + NO_2_^−^ and similarly, the short lifetime of ONOOH, rapidly loses its efficacy over time. pH and initial concentrations may need to be adapted to function under given conditions.

## Figures and Tables

**Table 1:** pH values of various phosphate-citrate buffer solutions with the peroxynitrous acid-containing solutions after step 2 (*Buffer + XmM H_2_O_2_ and NaNO_2_*) and after step 3 (addition of CASO broth to solution from step 2).

Sample	pH of Buffer + 25 mM H_2_O_2_ and NaNO_2_	pH of Buffer + 0.05 mM H_2_O_2_ and NaNO_2_	pH of Buffer + 25 mM H_2_O_2_ and NaNO_2_ + CASO	pH of Buffer + 0.05 mM H_2_O_2_ and NaNO_2_ + CASO	pH Employed in Numerical Calculations
1	2.39	2.39	2.39	2.39	2.4
2	2.75	2.85	2.84	2.91	2.8
3	3.23	3.25	3.26	3.27	3.2
4	3.76	3.76	3.78	3.78	3.8
5	4.18	4.23	4.26	4.30	4.2
6	4.73	4.76	4.85	4.86	4.8
7	5.46	5.50	5.51	5.54	5.5
8	5.93	5.71	5.95	5.73	5.9

**Table 2:** cfu per plate of individual measurements (M1 to M3) after treatment with 4-Nitroquinoline-*N*-oxide (100% = 100 µg per plate) or ONOOH-solution (100% = the initial concentration in mixed solution amounted to 25 mM NaNO_2_, 25 mM H_2_O_2_, 1% citric acid).

Test Product	Amount of Test Product	M1	M2	M3	Mean ± σ
4-Nitrochinolin-*N*-Oxid	100%	0	0	0	0.0 ± 0.0
	10.0%	35	0	21	18.7 ± 17.6
	5.0%	152	108	105	121.7 ± 26.3
	2.5%	160	172	174	168.7 ± 7.6
	1.0%	32	31	28	30.3 ± 2.1
	0.50%	13	12	11	12.0 ± 1.0
	0.30%	7	5	16	9.3 ± 5.9
H_2_O_2_ + NO_2_^−^	100%	0	0	0	0.0 ± 0.0
	20.0%	0	0	0	0.0 ± 0.0
	10.0%	7	9	11	9.0 ± 2.0
	4.0%	12	9	12	11.0 ± 1.7
	2.0%	12	11	6	9.7 ± 3.2
	0.40%	12	11	14	12.3 ± 1.5
